# Increased Production of IL-4 and IL-12p40 from Bronchoalveolar Lavage Cells Are Biomarkers of *Mycobacterium tuberculosis* in the Sputum

**DOI:** 10.1371/journal.pone.0059461

**Published:** 2013-03-20

**Authors:** Anna Nolan, Elaine Fajardo, Maryann L. Huie, Rany Condos, Anil Pooran, Rodney Dawson, Keertan Dheda, Eric Bateman, William N. Rom, Michael D. Weiden

**Affiliations:** 1 Division of Pulmonary, Critical Care, and Sleep Medicine, New York University School of Medicine, New York, New York, United States of America; 2 Lung Infection and Immunity Unit, CTBRI, Division of Pulmonology, Department of Medicine, University of Cape Town and Groote Schuur Hospital, Cape Town, South Africa; San Francisco General Hospital, University of California San Francisco, United States of America

## Abstract

**Background:**

Tuberculosis (TB) causes 1.45 million deaths annually world wide, the majority of which occur in the developing world. Active TB disease represents immune failure to control latent infection from airborne spread. Acid-fast bacillus (AFB) seen on sputum smear is a biomarker for contagiousness.

**Methods:**

We enrolled 73 tuberculosis patients with extensive infiltrates into a research study using bronchoalveolar lavage (BAL) to sample lung immune cells and assay BAL cell cytokine production. All patients had sputum culture demonstrating *Mycobacterium tuberculosis* and 59/73 (81%) had AFB identified by microscopy of the sputum. Compared with smear negative patients, smear positive patients at presentation had a higher proportion with smoking history, a higher proportion with temperature >38.5^0^ C, higher BAL cells/ml, lower percent lymphocytes in BAL, higher IL-4 and IL-12p40 in BAL cell supernatants. There was no correlation between AFB smear and other BAL or serum cytokines. Increasing IL-4 was associated with BAL PMN and negatively associated with BAL lymphocytes. Each 10-fold increase in BAL IL-4 and IL-12p40 increased the odds of AFB smear positivity by 7.4 and 2.2-fold, respectively, in a multi-variable logistic model.

**Conclusion:**

Increasing IL-4 and IL-12p40 production by BAL cells are biomarkers for AFB in sputum of patients who present with radiographically advanced TB. They likely reflect less effective immune control of pathways for controlling TB, leading to patients with increased infectiousness.

## Introduction

The global plan for tuberculosis (TB) by the Stop TB Partnership is to halve the prevalence and mortality caused by TB by 2015, followed by eliminating TB as a public health problem by 2050. [1] Tuberculosis is an infection spread through the air. TB patients frequently cough and generate aerosols of *Mycobacterium tuberculosis (Mtb)* that dry and produce suspended fomites. Once inhaled, the *Mtb* lodges in an unexposed person’s lung, rehydrates and starts to replicate slowly. A TB specific Th1 immune response develops, preventing active tuberculosis but failing to eradicate all viable *Mtb*, producing latent tuberculosis infection (LTBI). Approximately 10% of HIV negative LTBI patients develop active TB over the course of their lives. With HIV co-infection, a large majority of those with LTBI will develop active disease [2]. In high TB burden countries like South Africa, approximately 40–50% of the population has LTBI and 1% of the population of South Africa developed active TB in 2009 producing an annual incidence of 480,000 cases [3].

The mycolic acid of *Mtb* cell walls appears bright red with Ziehl-Neelsen stain that is also called acid fast stain. *Mtb* is the most common acid fast staining bacillus (AFB). Observing AFB on microscopic examination of the sputum in a patient with pulmonary infiltrates and cavities is consistent with tuberculosis; culture of *Mtb* and/or molecular diagnostics confirms the diagnosis. While AFB staining is less sensitive and specific than *Mtb* culture, the AFB test yields results in hours compared to the weeks required for *Mtb* culture. In addition, the AFB smear is also useful for measuring infectiousness. A patient with AFB smear negative, culture positive TB has a low probability of transmitting infection compared to a patient with smear positive TB [4], [5].

The CD4^+^ Th1 lymphocyte response is required for effective acquired immunity to *Mtb* [*6*]. Adding the Th1 cytokine interferon gamma by aerosol improves response to therapy by more rapidly converting the sputum smear from positive to negative and reducing respiratory symptoms [7]. Alternatively, patients with mutations in Th-1 cytokine signaling pathways such as interferon gamma and IL-12 (a p40 and p35 heterodimer) are susceptible to overwhelming infection with *Mtb* [8], [9]. Impaired CD4^+^ Th1 lymphocyte response in HIV infection also produces ineffective immunity to *Mtb* [*2*]. Several observations suggest that the Th2 cytokines, IL-4 and IL-10, are associated with LTBI reactivation and advanced TB [10]–[13]. The antagonist splice variant IL-4delta-2 reduces the risk of severe disease and reduced the risk of progression to active disease in contacts of patients with active disease [14]–[16]. Surprisingly, an IL-12p40 homodimer is expressed in Th2 disease such as asthma [17].

To better understand the relation of immune state to TB transmissibility in HIV negative patients who presented with culture confirmed TB and extensive infiltrates/cavities, we studied immune cells from the lower respiratory tract using BAL. We performed a cross sectional study of an urban cohort in Cape Town presenting with extensive infiltrates and many with at least one cavity. We observed that increasing release of IL-4 and IL-12p40 by BAL cells were risk factors for being AFB smear positive. These results suggest immune pathways that are Th2-like with increased IL-4 and IL-12 p40 release are associated with increased infectiousness.

## Methods

Study Subjects: Patients with pulmonary tuberculosis were recruited from April 2005 to December 2006 in the Division of Pulmonology at the University of Cape Town with the following inclusion criteria [7]: All patients had *Mtb* cultured from their sputum, were drug-sensitive and had extensive infiltrates. Age, gender, race/ethnicity, fever, respiratory symptoms, and smoking status were obtained at screening evaluation. BMIs were calculated from height and weight measured at screening. Sputum smear was performed at the time of bronchoscopy two weeks after screening. IRB approval was obtained at NYU School of Medicine and the University of Cape Town; all study subjects signed informed consent in their native language.

Bronchoalveolar lavage (BAL): BAL was performed after local anesthesia with lidocaine; the bronchoscope was inserted via the nasal passage to the lower respiratory tract, wedged, and a 300-ml lavage was performed using 5, 20-ml aliquots of normal saline in each of three involved lung segments. The recovered fluid was pooled and filtered over sterile gauze, and a total cell count performed. A cytospin slide was stained with Diff-Quik and 500 cells counted to determine the cell differential. BAL supernatants were cultured in RPMI over 24 hours at 10^6^ BAL cells/ml. BAL cell supernatants were thawed once at 4°C and assayed using 13-Plex Human Pro-inflammatory Panel according to manufacturer’s instructions (Millipore, Billerica, MA). The supernatants were analyzed on a Luminex 200IS (Luminex Corporation, Austin, TX) using MasterPlex _TM_ QT software (Ver. 1.2; MiraiBio, Inc).

Statistical Analysis: Database management and statistics were performed using SPSS 20 (IBM, Armonk, NY). Normally distributed data were expressed as mean and standard deviation. Data not distributed normally and multi-analyte comparisons were evaluated using a Mann Whitney U-test. Hierarchical clustering was performed using furthest neighbor squared Euclidean distance with Z-score transformed variables. Analytes that were significantly different between smear positive and negative were used to construct logistic regression models with AFB smear status as the outcome variable. The models were adjusted for potential confounders that were chosen after the study was completed. BMI was chosen because malnutrition is strongly immune-compromising, smoking status was significantly different between smear positive and smear negative patients. Gender and race were added because of different potential differences in environmental exposures. Significance was assessed at p<0.05.

## Results

By design all 73 patients in this cohort had culture positive pulmonary tuberculosis with extensive infiltrates on their chest x-rays. The group was young with a median age of 34 years and was 75% male. Black Africans accounted for 41% of the cohort while patients reporting more than one race accounted 59%. They were underweight with a median BMI of 18.7. A large proportion (77%) had a history of current smoking. They uniformly had reactivity to tuberculin skin testing (25 mm). A minority (41%) had fever on presentation or cavities on chest x-ray (31%) (Table 1).

**Table 1:** Demographics.

		AFB Smear		P
		+	−	
N		59	14	
Age*		35 (11)	30 (9)	0.118
Gender (Male)†		45 (76.3)	10 (71.4)	0.705§
BMI*		19.01 (2.55)	18.76 (2.54)	0.747§
Smoking (Ever)†		49 (83.1)	7 (50)	0.009
Race†	Black	24 (40.7)	6 (42.9)	
	>1	35 (59.3)	8 (57.1)	0.882
	Other**	0	0	
TST (mm)*		32 (15)	28 (10)	0.257§
Cavities on CXR		17 (28)	5 (36)	0.613
Fever†		28 (47.5)	2 (14.3)	0.023

* Mean (SD);

† N(%).

§ unpaired t-test with Welch’s correction; all other significance assessed by chi-squared;

** Other-Asian/White/Unknown.

Our primary outcome was bacterial burden as assessed by AFB on sputum smear. At presentation 59/73 (81%) had high bacterial burden (smear positive) with AFB observed on sputum microscopy and 14/73 (19%) had low bacterial burden with no AFB observed in sputum (smear negative). Those with fever and ever smokers were more likely to be AFB smear positive (48% vs. 14%, p<0.03 and 83% vs. 50%, p<0.01 respectively). There was no significant association between smear status and age, gender, BMI, race, tuberculin skin test (TST) size, or the presence of cavities on chest x-ray (Table 1).

We assessed the immune response using BAL cell differential and cytokine elaboration after 24 hours of ex vivo culture. AFB smear positive patients had higher total BAL cells/ml (19.3 vs 15.6 x10^3^/ml, p<0.03, Table 2). Alternatively AFB smear positive patients had lower proportion of BAL lymphocytes (4% vs 10%, p = 0.002, Table 2).

**Table 2:** BAL Cell Differential at Baseline.

	AFB Smear		P
	+	−	
N	59	14	
Cells/mL x 10^4^	19.3 (12–33)	15.5 (6–17)	0.027
AM,%	60 (19–80)	62 (52–77)	0.330
Lymph,%	4 (2–8)	10 (6–15)	0.002
PMN, %	31 (10–79)	25 (9–38)	0.282
Eos, %	–	1 (1–1)	–

Data represented as Median(IQR) and significance measured by Mann-Whitney U.

**Figure 1:**
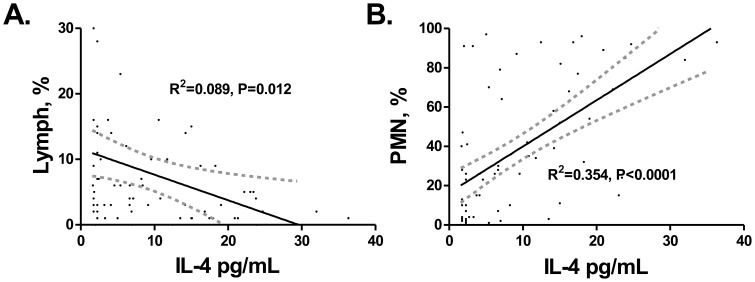
Correlation of BAL supernatant IL-4 with BAL cell differential. A. Lymphocyte %. B. Polymorphonuclear Neutrophils %. Linear regressions with 95% confidence intervals.

Cytokine elaboration in 24-hour culture supernatants was measured by multiplex bead fluorescence assay. AFB smear positive patients versus smear negative patients had significantly higher IL-4 and IL-12p40 production (7.68 pg/ml vs. 2.6 pg/ml p<0.01 and 102 pg/ml vs. 3.2 pg/ml p = 0.01, Table 3 respectively). There was a trend to higher IL-10 elaboration in smear positive patients (8.95 pg/ml vs. 6.09 pg/ml p = 0.068, Table 3). There was no significant difference in IL-6, IL-8, TNF-α, IP-10 or IFN-γ elaboration between smear negative and smear positive patients. There was a significant negative correlation between BAL lymphocytes and IL-4 production (R^2^ = 0.09, p = 0.012). In addition, there was a strong positive correlation between BAL PMN and IL-4 (R^2^ = 0.354, p<0.0001, Figure 1). To assess the contribution of environmental exposures to IL-4 production we cultured BAL cells from 8 patients recruited from Cape Town with clear chest x-rays. In the absence of stimulation, none had measurable IL-4 levels using a high sensitivity IL-4 ELISA with a limit of detection 0.25 pg/ml. As a positive control the cells were treated with RD-1; after stimulation eighty seven percent 7/8(87%) had IL-4 above the limit of detection with a mean level of 8.2±7.6pg/ml There was no significant correlation between BAL cell differential and IL-6, IL-8, IL-10, TNF-α, IP-10 or IFN-γ. There was no correlation between BAL cell cytokine production and serum cytokine levels (data not shown).

**Table 3:** BAL Analytes based on AFB Smear Status at Presentation.

Analyte	Smear		P
	+	−	
IL-4	7.68 (2.3–16.8)	2.57 (1.7–6.2)	0.009
IL-6	5.89 (4.0–30.7)	5.95 (4.0–8.2)	0.597
IL-8	591.08 (230–2989)	448.5 (181–1060)	0.516
IL-10	8.95 (5.6–15.2)	6.09 (3.4–6.1)	0.068
IL-12(p40)	101.7 (3.2–299)	3.20 (0.6–75.3)	0.010
TNF-α	11.04 (4.5–39.1)	12.63 (3.5–25.4)	0.961
IP-10	24.39 (14.9–50.4)	19.36 (10.4–32.9)	0.408
IFN-γ	10.43 (6.0–18.5)	3.77 (2.7–11.4)	0.111

Analytes are in Median (IQR); pg/mL.

Mann-Whitney U used to test of Significance.

**Figure 2:**
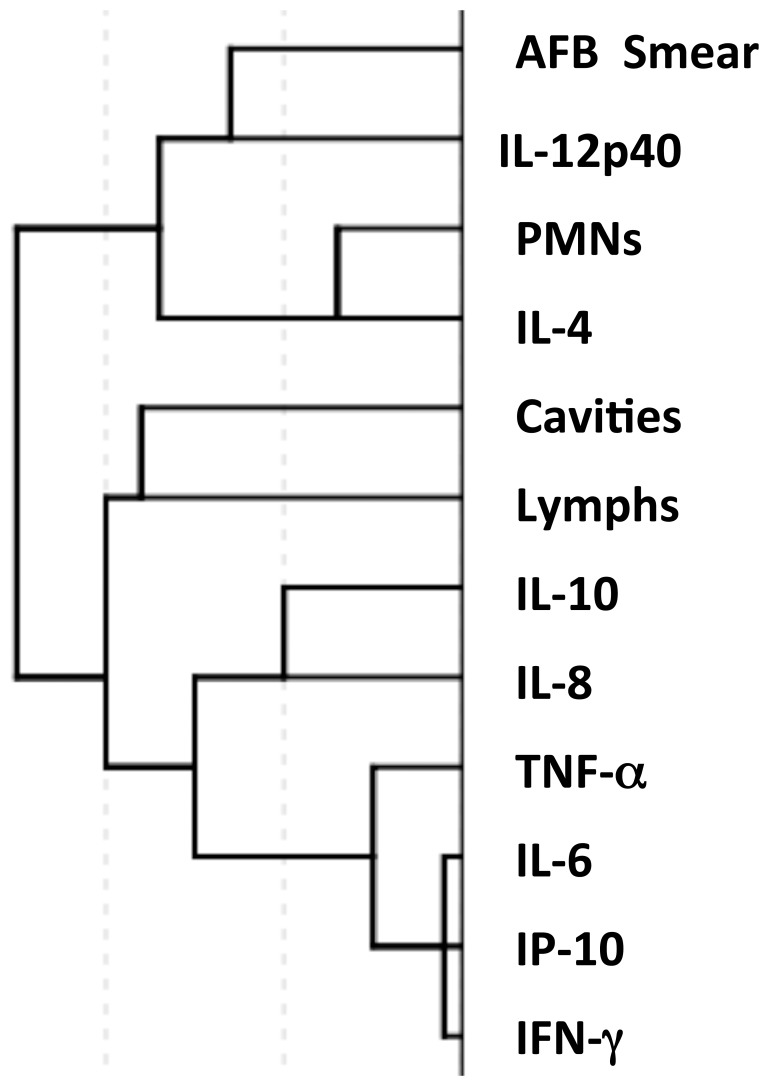
Hierarchical clustering of results from sputum smear for AFB, BAL cell differential, BAL cell culture supernatants and cavities on chest radiograph demonstrates AFB smear clusters with IL-4, IL-12 p40 and BAL PMN.

We used hierarchical clustering to determine the association between immunologic, radiographic findings and AFB smear status. AFB observed in induced sputum clustered with IL-4 and IL-12p40. Consistent with the results of the linear regression, IL-4 and PMN clustered together (Figure 2). Even though there was trend towards increased IL-10 in smear positive patients, it did not cluster with the AFB smear. Interestingly, the Th-1 cytokines IFN-γ and IP-10 clustered together with TNF-α, another cytokine important for effective TB immunity.

We then tested the ability of BAL cell cytokines to predict the presence of AFB on examination of sputum smear (Table 4). Each 10-fold increase in IL-4 significantly increased the odds of AFB in the sputum by 7.4-fold (95% CI 1.2–46) in a logistic model adjusted for BMI, ethnicity, gender and smoking. Each 10-fold increase in IL-12p40 also increased risk for positive AFB smear by 2.2-fold (95% CI 1.1–4.3). There was no association between IL-10 and smear status (95% CI 0.45–23) (Table 4).

**Table 4:** Regression Analysis for AFB smear positive.

Analyte	OR	95% CI
IL-4	7.382	1.19–45.97
IL-12 (p40)	2.176	1.09–4.34

Analytes pg/mL Log_10._

Adjusted for BMI, Ethnicity, Gender and smoking Status (Ever/Never).

Hosmer-Lemeshow (Goodness of Fit) Sig. >0.05 for each single analyte model.

## Discussion

We performed a cross sectional study of cytokine release into 24-hour BAL supernatants on 73 HIV-negative urban South African patients who presented with pulmonary tuberculosis. All had extensive infiltrates on chest x-ray and a significant minority had cavities. They were severely underweight with median BMI of 18.9, but maintained active antigen-specific cellular immunity to *Mtb* with strongly reactive tuberculin skin tests. The study cohort represents young adults, a group whose reproductive and economic potential is negatively impacted by TB. They put contacts at home and at work at risk for *Mtb* exposure. An improved understanding of the risk factors for transmission is therefore important. The infectivity of pulmonary tuberculosis was ably described by Shaw and Wynn-Williams in London six decades ago when smear positive cases resulted in 65% contact conversion of tuberculin tests in children 0–14 years whereas smear negative culture positive cases resulted in 27% conversion and smear negative culture negative was 18% [4]. They reported that 54/374 children who were close contacts of smear positive source cases of tuberculosis developed active tuberculosis, compared to 6/228 who were close contacts of smear negative and culture positive patients (OR = 6.2, 95% CI 2.6–14.7).

In addition to routine clinical evaluation, we performed research bronchoscopy to retrieve lung immune cells from the site of infection. This allowed us to assess cytokine production and BAL cell differential in association with sputum AFB smear status. We observed that each 10-fold increase in production of the Th2 cytokine IL-4 increased the odds of being smear positive by 7.4-fold. IL-10, another Th2 cytokine that is a TB biomarker, was not a significant predictor of sputum AFB smear status in this cohort suggesting that IL-4 is the predominant Th2 biomarker that predicts poor TB control. Cytokine co-expression in human Th1/Th2 cells differentiates in response to GATA3, T-bet, and other transcription factors, with IL-4, IL-5, and IL-13 regulated by a coordinated mechanism whereas IL-10 is expressed by a different subset of cells that is prevalent at early stages of Th2 differentiation [18]. Increased IL-4 gene expression in PBMCs has been found in non-Beijing strains of TB associated with more cough, fever, night sweats, advanced radiological disease, and cavities [19]. Increased CD8+IL-4 T cells predicted progression from latent infection to active disease in 6/10 health care workers [20]. We did not measure the IL-4δ2 splice variant which is an IL-4 antagonist and is increased in BAL cells in TB/HIV co-infection [14], [15].

IL-4 has been implicated in conversion of LTBI to active TB. It has also been associated with radiographic extent of disease but not AFB smear status [10]. We now report a strong positive association between BAL-cell IL-4 production and the presence of AFB on sputum smear. There was no association between BAL-cell IL-4 and serum IL-4 levels suggesting the pulmonary inflammation during TB is not accurately reflected in the systemic compartment or venous blood. There was a negative association between BAL IL-4 and BAL lymphocytes. This could underlie the impact of IL-4 on sputum AFB since smear positive patients have lower BAL lymphocytes than smear negative patients. Further investigation of the impact of IL-4 production on lymphocyte number and function in tuberculosis is needed to better understand this observation. While smear status did not correlate with PMN in the BAL, there was a strong positive linear correlation between IL-4 and PMN in BAL. The proportion of patients with elevated PMN in this cohort is noteworthy. Half the patients had more than 25% PMN in their BAL cell differentials. Only the lowest quartile had fewer than 10% PMN in their BAL. *Mtb* inhibits both macrophage and PMN apoptosis leading to delayed activation of naïve CD4 cells [21]. We previously noted that IL-8 in BAL fluid correlated with PMN in BAL from TB patients [22], but we did not replicate this in BAL supernatants. IL-4 has been postulated as a key player in TB pathogenesis, especially with its ability to down-regulate inducible nitric oxide synthase, Toll-like receptor 2, and macrophage activation [23]. Flow cytometric analysis of IL-4 production in CD8 and CD4 cells in blood from TB patients showed increases in patients with cavities although we did not find increased BAL supernatant IL-4 in our TB patients with cavities [24]. IL-4 mRNA in PBMC has been associated with advanced pulmonary TB as well as a subgroup with increased antagonist IL-4δ2; the role of Th2 cytokines in human tuberculosis requires further investigation [10].

We also observed that smear positive patients had higher IL12 p40 than smear negative patients (101 pg/ml vs 3.2 pg/ml p<0.01). Each 10-fold increase in IL-12p40 production increased the odds of being smear positive by 2.2-fold. The low level of IL-12p40 production by BAL cells has been observed in patients with minimal TB from Chandigarh, India [25] and in volunteers with normal chest x-rays in New York [26]. The elevated BAL cell production of IL-12p40 appears to occur during high burden disease.

The observation that elevated IL-12p40 was a risk factor for being smear positive was surprising since IL-12 is a Th1 cytokine important for TB control in humans [8]. IL-12 is a heterodimer made up of a p40 and p35 subunit. The p40 subunit has other binding partners that lead to cytokines with varied functions. A possible explanation for the finding is that IL-12p40 homodimers form and function as a macrophage chemo-attractant as well as a competitive antagonist of IL-12 [17], [27], [28]. In vivo IL-12p40 homodimers are elevated in asthma, a disease characterized by Th2 hyperactivity. Our immunoassay did not give information on the binding partner of IL-12p40 in this setting requiring further investigation for a better understanding of this observation. Immunohistochemistry of TB granulomas consistently showed IL-12p40 in CD8+ cells; 75% of granulomas were positive for both IFN-γ and IL-12, and 4/7 TB patients expressed IL-4 and IFN-γ in granulomas [29]. IL-12p40 is required for dendritic cell migration and T cell priming after *Mtb* infection in transgenic IL-12p40^−/−^ mice [30]. IL-12^−/−^ mice infected by airways inoculation of BCG do not have macrophages, lymphocytes, or Th1 cytokine responses, but do not have a Th2 response either and succumb to local growth and systemic spread of bacilli [31].

A history of smoking, oral temperature greater than 38.5°C upon enrollment and high BAL cell numbers were significantly associated with being AFB smear positive. Nutritional state as measured by BMI was not different between smear negative and smear positive patients. The negative effects of smoking on lung immunity in tuberculosis are well documented and demonstrated in this study [32]. A systematic review and meta-analysis of 43 observational studies found smokers compared to people who do not smoke, have an increased risk of a positive TST, of having active TB (summary estimate of 2.01), and of dying of TB [32]. There were additional risks from passive cigarette smoke and indoor air pollution from biomass burning. Smoking also contributes to increased neutrophils in the BAL. Fever and increased BAL cell concentration are non-specific markers on inflammation. It is not surprising that patients with higher *Mtb* burden have increased manifestations of inflammation. Tracey’s group has shown that acetylcholine-producing memory T cells activated by the vagus nerve via the α7-nicotinic acetylcholine receptor inhibit TNF-α production in the spleen, and nicotine in cigarette smoke can target the same receptor potentially inhibiting TNF-α production, which is essential for protective host immunity in TB [33].

This study has several limitations. It is a cross sectional natural history investigation and the observed associations do not imply causality. In addition, the findings on BAL-cell cytokine production are unlikely to be clinically useful since they require invasive and expensive testing. Finally, there are likely to be cohort specific effects on our results; these findings need to be replicated in other cohorts to assess their generalizability. Nevertheless, these human observations are important to inform more mechanist investigations on the Th2 response to tuberculosis in animal models.

Another important line of investigation informed by these results is to develop non-invasive screening tests for Th2 predominant response in TB. Such a test could stratify the risk of transmission; a critical issue with MDR-TB. Some possibilities include cytokine measures on expectorated sputum or cytokine elaboration in blood stimulated with TB antigens. It date, interferon gamma release assays are clinically useful for assessing TB exposure. IL-4 or IL-12p40 release assays done in parallel may add important information that can prioritize patients for chemoprophylaxis.

### Conclusion

Increased production of the Th2 cytokine IL-4 by BAL cells is a strong risk factor for TB transmission in this South African cohort. Further investigation of Th2 immune pathways in TB are needed to improve our understanding of immune states that predispose to TB transmission and develop a clinical useful screening test for a Th2 predominant immune response.
